# Enhancing Stability and Tooth Bleaching Activity of Carbamide Peroxide by Electrospun Nanofibrous Film

**DOI:** 10.3390/ph13110381

**Published:** 2020-11-11

**Authors:** Siriporn Okonogi, Adchareeya Kaewpinta, Thomas Rades, Anette Müllertz, Mingshi Yang, Sakornrat Khongkhunthian, Pisaisit Chaijareenont

**Affiliations:** 1 Department of Pharmaceutical Sciences, Faculty of Pharmacy, Chiang Mai University, Chiang Mai 50200, Thailand; 2 Research Center of Pharmaceutical Nanotechnology, Chiang Mai University, Chiang Mai 50200, Thailand; sakornratk@hotmail.com (S.K.); yodent@hotmail.com (P.C.); 3 Interdisciplinary Program in Nanoscience and Nanotechnology, Faculty of Science, Chiang Mai University, Chiang Mai 50200, Thailand; akaewpinta@gmail.com; 4 Department of Pharmacy, Faculty of Health and Medical Sciences, University of Copenhagen, 2100 Copenhagen, Denmark; thomas.rades@sund.ku.dk (T.R.); anette.mullertz@sund.ku.dk (A.M.); mingshi.yang@sund.ku.dk (M.Y.); 5 Wuya College of Innovation, Shenyang Pharmaceutical University, Shenyang 110016, China; 6 Department of Restorative Dentistry and Periodontology, Faculty of Dentistry, Chiang Mai University, Chiang Mai 50200, Thailand; 7 Department of Prosthodontics, Faculty of Dentistry, Chiang Mai University, Chiang Mai 50200, Thailand

**Keywords:** electrospun nanofibers, carbamide peroxide, water soluble drug, tooth bleaching, drug delivery, controlled release

## Abstract

Carbamide peroxide (CP) possesses a strong tooth bleaching activity, however, its clinical application is limited because of its instability in aqueous formulations. This study explores the improvement of CP stability and bleaching activity by loading CP in electrospun nanofibrous film (ENF). Polyvinylalcohol, polyvinylpyrrolidone, and silica were used as components for core-based nanofibers of ENF. Electrospinning feed aqueous solutions (EFASs) were developed for preparing CP loaded ENF (CP-ENF). Stability of CP in EFASs is significantly higher than in pure water. The highest stability of CP is found in PPS-CP3, composed of 0.5% CP, 5.5% polyvinylalcohol, 3% polyvinylpyrrolidone, and 1% silica. The results from X-ray diffraction indicate that CP is dispersed as a non-crystalline form in CP-ENFs. CP and the compositions of EFASs play a major role on characteristics and bleaching efficiency of CP-ENFs. Drug release of CP-ENFs is the first order kinetics. CP-ENF obtained from PPS-CP3 shows the highest drug entrapment efficiency, high adhesion, and suitable sustained release. Drug release mechanism is along with anomalous transport according to Korsmeyer–Peppas model. In an ex vivo study using human teeth, it shows the highest bleaching efficiency among the others. Therefore, CP-ENF obtained from PPS-CP3 is a promising ENF for clinical use.

## 1. Introduction

Hydrogen peroxide and carbamide peroxide (CP) are strong oxidizing agents and have been used as tooth bleaching drugs for a long time [1]. Hydrogen peroxide however, easily decomposes. It can react with any present oxidizable compound and catalytic agents resulting in the formation of oxygen and water [2]. CP is known as urea peroxide or hydrogen peroxide-urea as it is composed of hydrogen peroxide and urea in a solid state and is more stable than hydrogen peroxide [3]. Therefore, CP is more commonly used than hydrogen peroxide as an active ingredient in tooth bleaching products [4,5]. High CP concentration of up to 35% can be found in bleaching products in in-office tooth bleaching treatment whereas concentrations of 5–22% are used in at-home tooth bleaching treatment [6,7]. At-home tooth bleaching has the advantage of self-administration by patients, and higher safety and fewer side effects due to the lower CP concentrations [8]. Previous reports showed that using low CP concentrations with long treatment time provides bleaching efficiency equivalent to high CP concentrations [9] and could decrease side effects such as tooth sensitivity, gingival irritation, and toxicity to pulp cells that can be caused by high peroxide concentrations [10].

CP is freely soluble in water and easily decomposes in aqueous or hydrophilic formulations, to hydrogen peroxide and urea. The formed hydrogen peroxide can further decompose to water and oxygen as mentioned above. Many antioxidants have been used as stabilizers in aqueous tooth bleaching formulations of CP for the purpose of preventing CP decomposition. However, the stabilizers have different disadvantages such as price, undesirable color, poor solubility, and lack of effectiveness. Therefore, formulations with low or no water content would be desired to deliver CP to the teeth for bleaching. In the past, CP loaded microspheres were developed. This system was prepared by an emulsification technique to generate a water-in-oil emulsion including shellac ammonium salt solution with soluble CP as an internal phase dispersed in a mixture of sunflower oil and calcium chloride powders as an external phase. In this process, gelation between shellac and calcium occurred and CP loaded microspheres were covered with a calcium-shellac gel [11]. However, rapid decomposition of CP was observed due to water remaining in the internal phase of the microspheres.

Nanofibers are solid nanomaterials with diameters in the range of 100–1000 nm [12]. Layered nanofibers or nanofiber films can be developed from different polymers and drugs can be incorporated into the fibers or the space between the layered fibers. Nanofibers have unique properties such as a large specific surface area and high porosity [13]. Nanofibers are applied in various fields of medical and pharmaceutical applications, e.g., as carriers for cell cultivation [14], medical prostheses, tissue engineering scaffolds [15], wound dressings, cosmetic, and drug delivery systems [16]. The technique of incorporation of active compounds into the nanofibers is crucial for each application [17]. Nanofibers can be prepared by techniques such as drawing [18], template synthesis [19], phase separation [20], self-assembly [21], and electrospinning [22]. Among these techniques, electrospinning is considered a method of choice as it is comparatively simple and capable of producing ultrafine micro- to nano-scale fibers [23]. The electrospinning equipment consists of three major components: a polymer solution reservoir with a syringe pump connecting to a spinneret needle, a grounded collector, and a high voltage power supply. When a strong electric field with a high voltage is applied, a spinning solution is pumped through the spinneret. With an increase in the voltage, electrostatic forces deform the droplet into a pointed shape. When the voltage exceeds a threshold value where the electric forces overcome the surface tension of the droplet, the solution is ejected from the tip of the droplet. As the spinneret needle moves towards a grounded collector, the solvent can be evaporated and nanofibers can be formed [24]. As the obtained nanofiber has low water content, it should be suitable for loading CP to retard CP degradation. To the best of our knowledge, there are no reports on nanofiber formulations of CP in the literature. The present study is the first investigation to develop CP loaded electrospinning nanofibrous films (CP-ENFs). The main aim of our study was to stabilize and enhance tooth bleaching activity of CP. Various formulations of electrospun feed aqueous solutions (EFASs) were developed for producing CP-ENFs which CP concentration of 5–10%. The effective EFASs were selected based on their electrospinnability for preparing CP-ENFs. Internal solid structure, morphology, adhesive property, drug entrapment efficiency, drug release properties, and ex vivo tooth-bleaching efficiency of the obtained CP-ENFs were investigated.

## 2. Results and Discussion

### 2.1. Preparations and Characteristics of EFASs

Polyvinylalcohol (PVA), polyvinylpyrrolidone (PVP), and silica were selected as major components in EFASs for preparing core-based nanofibers of ENFs because of their desirable properties. PVA possesses highly favorable characteristics such as good swelling, bioadhesive, biocompatible, and biodegradable properties under both aerobic and anaerobic conditions [25]. Certain concentration of PVA, approximately 4–10% in water, was used in EFASs in the present study. For PVP, it has excellent water solubility with good electrospinnability and is compatible with a wide range of hydrophilic materials [26,27]. Previous studies have shown that PVP can stabilize hydrogen peroxide by forming strong hydrogen bonds [28]. In addition to these two polymers, silica was included in the formulations as it is an inert material with a large surface area for drug adsorption, which may enhance the drug entrapment efficiency [29]. Mixing these excipients at different concentrations with CP in water could yield six effective EFASs for preparing CP-ENFs as shown in Table 1. CP-EFASs were subjected to the electrospinning process, the water evaporated, and CP-ENFs formed. This study aimed to load CP in ENFs at low concentrations of 5–10%; therefore, CP concentrations of 0.5–1% in EFASs were used. EFAS without CP such as P-BL was used as a blank or negative control.

Viscosity and conductivity of EFASs are two important physical properties that influence the electrospinnability [30]. Our results as shown in Table 2 demonstrate the viscosity and conductivity of EFASs. The result of the present study indicates that polymer concentration plays a significant role on the viscosity of EFASs. For example, in P-BL which contains 10% PVA and without CP, showed significantly higher viscosity than P-CP, the solution contained 9% PVA and 1% CP. This result indicates that only 1% decrease of PVA concentration can cause a significant decrease of EFAS viscosity. The conductivity of P-BL is lower than that P-CP, indicating that low concentration of PVA can cause high conductivity of the solution. Replacing 3% PVA of P-CP with PVP yielded PP-CP and this led to an increase in the viscosity and conductivity of the solution. This result demonstrates the effects of PVP on the viscosity and conductivity of the system. However, replacing 1% PVA of P-CP with silica yielded PS-CP and this led to a decrease in viscosity whereas the conductivity was not altered. It was previously reported that silica could break down the gelling structure of the polymer [31]. Therefore, adding silica resulted a decrease the viscosity of EFASs. Effects of CP and silica on conductivity can be clearly seen in EFASs containing PVA, PVP, silica, and CP, i.e., PPS-CP1, PPS-CP2, and PPS-CP3. Replacing 0.5% PVA of PPS-CP3 with CP yielded PPS-CP1 with lower viscosity but this did not affect conductivity. Replacing 1% PVA of PPS-CP1 with silica yielded PPS-CP2 and this led to a decrease in viscosity and conductivity. The decrease in the conductivity is due to the fact that silica has a poor electrical conductivity [32].

### 2.2. Stability of CP in EFASs

**Figure 1:**
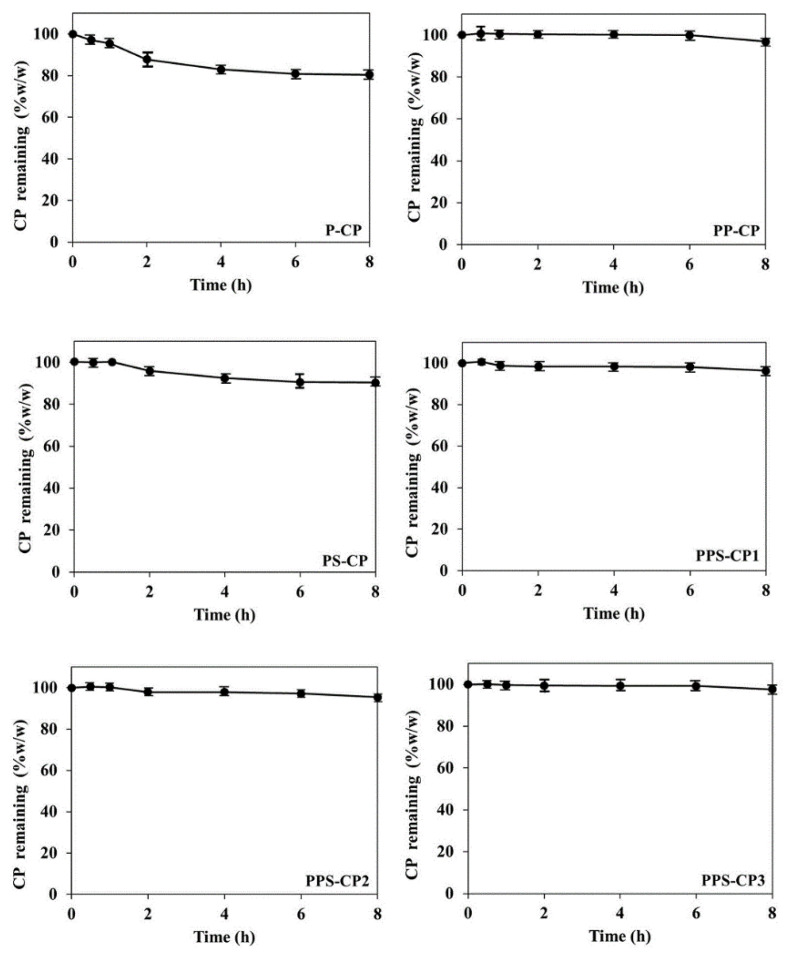
Stability profiles of carbamide peroxide (CP) in various EFASs at 25 °C.

Interaction of CP and the excipients in EFASs may occur and can lead to the loss of CP during fabrication of ENFs. Therefore, the stability of CP in EFAS is crucial before selection of a suitable EFAS. The remaining CP in each EFAS over a period of 8 h at 25 °C is shown in Figure 1. It is seen that the stability of CP in P-CP is the lowest in comparison among the others. Previous studies have shown that PVA is oxidized by a strong oxidant such as peroxide [33,34]. Oxidation of PVA in the presence of peroxide takes place via an initial cleavage of 1,2-glycol units in the PVA chain [35]. Thus, the rapid degradation of CP in P-CP is considered likely due to the oxidation reaction between PVA and CP. Replacing some part of PVA with silica, such as in PS-CP, yielded better CP stability. Silica is an inert material with a large surface area suitable for drug adsorption [29]. CP molecules might have been adsorbed to the silica and thereby are partly protected from direct contact with PVA. Blending PVA with PVP, for example in PS-CP and P-CP, also shows significant difference in improvement of CP stability. PVP can act as a good stabilizer and retard CP degradation because the carbonyl oxygen atoms of PVP can form hydrogen bonds with hydrogen peroxide molecules in the solution [28]. Therefore, replacing 3% PVA in P-CP with PVP yielded PP-CP and led to significantly higher stability of CP. The result of the present study also indicates that the potential of PVP on enhancing CP stability is much more effective than silica. This effect can be clearly seen when comparing CP stability in PP-CP (without silica) and PPS-CP1 in which 1% PVA in PP-CP was replaced with silica. It is found that in these two formulations with the same amount of PVP and different amount of silica, the enhancing stability of CP is similar.

### 2.3. Fabrication and Characterization of ENFs

**Figure 2:**
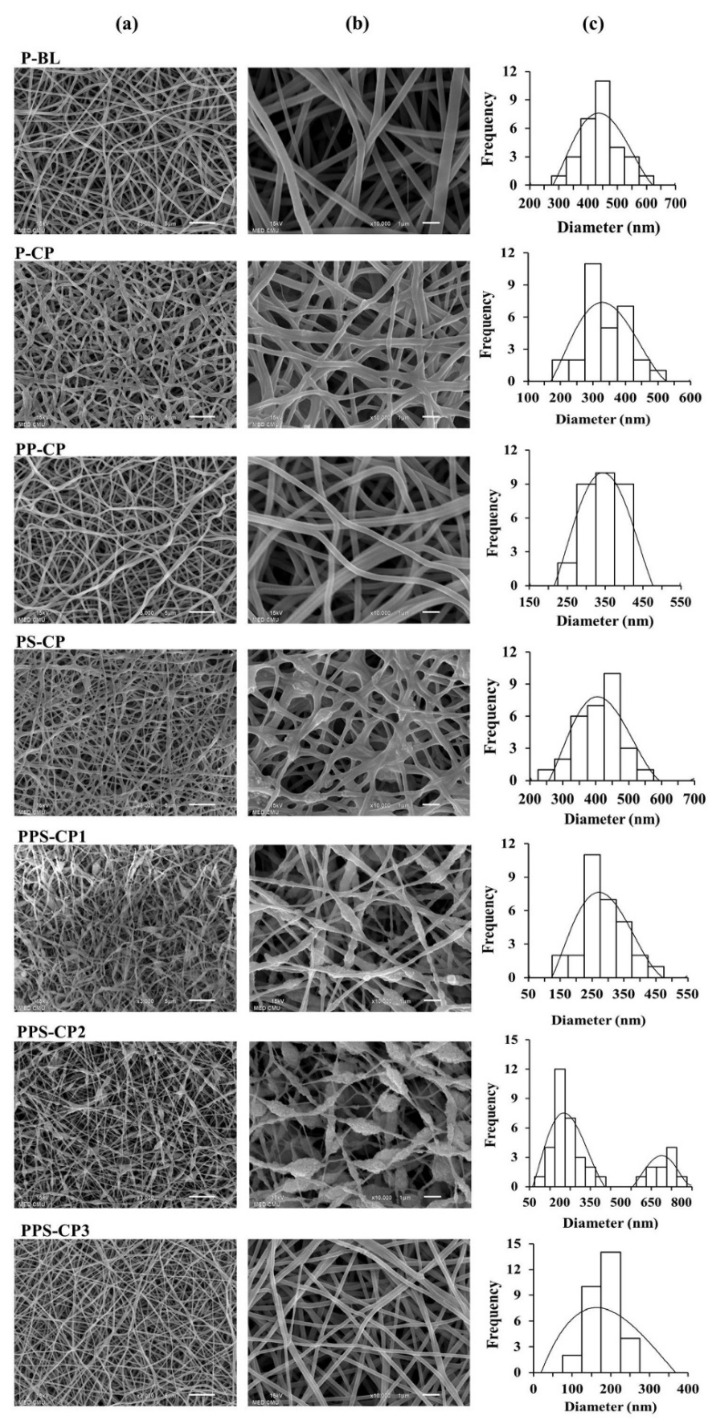
SEM images at magnifications of 3000× (**a**) and 10,000× (**b**) and size distribution (**c**) of the electrospun nanofibrous films (ENFs) obtained from various EFASs.

ENFs from the developed EFASs were successfully prepared via the electrospinning process employed in this study. The obtained ENFs are white and possess a smooth surface appearance. The scanning electron microscope (SEM) images, as shown in Figure 2, reveal that the nanofibers obtained from P-BL, a PVA solution without drug, is a non-woven structure with an average nanofiber diameter of 421 ± 68 nm. It is noted that after an addition of CP, as in P-CP, some parts of the fibers are merged and overlapped. The diameters of the nanofibers from P-CP are close to that of PP-CP with average values of 319 ± 69 nm and 321 ± 45 nm, respectively. The fibers of PP-CP are smoother than those of P-CP. This result is due to the ejected solution of PP-CP being a more homogenous and continuous jet than that of P-CP. Addition of silica to the feed solution can increase in the diameter of the fibers. This can be seen in PS-CP fiber that its average diameter is 401 ± 54 nm, significantly larger than that the fibers obtained from P-CP. PVP has been used as a polymer carrier and mixed with various compounds that are difficult to spin via electrospinning [36]. Our results show that the addition of PVP, at the expense of PVA, to the solutions containing PVA and silica, as in PPS-CP1, can yield fine nanofibers with smooth surfaces and an average diameter of 261 ± 73 nm. Increase silica content to the formulations, as in PPS-CP1 with PPS-CP2, some beaded nanofibers can be seen. Interestingly, the beaded nanofibers are absent in PPS-CP3 samples, and the obtained fibers are very fine with an average diameter of 159 ± 33 nm. Comparing PPS-CP2 and PPS-CP3, both solutions have the same ratio of silica to drug (2:1 *w*/*w*), however PPS-CP2 has a lower polymer concentration than PPS-CP3 leading to an obvious decrease in viscosity. This effect can influence the formation of highly beaded fibers from PPS-CP2. From these results, it is postulated that besides the excipient type, the concentration of the excipients is one of the important factors that influences the transformation of the feed solutions into nanofibers. It has been reported that a low polymer concentration can result in electrosprayed particles or beaded fibers, whereas a high polymer concentration can limit the fiber production because of too high viscosity [37]. In the present study, with a working voltage of 10 kV and a spinning distance of 10 mm, it is found that a concentration of 5–10% PVA solution is the most suitable for fiber production and no fibers could be collected when the PVA concentration was <4%. Therefore, the obtained nanofibers contained at least 50% of PVA and the highest amount of CP that could possibly be loaded in EFASs is 50%. However, from preliminary development of 50% CP loaded nanofibers, it was found that the obtained CP-ENF was very brittle and easily tears due to the low amount of polymer (data not shown). The reduction in the fiber diameter of the formulations containing CP and PVP can be attributed to the increase in the conductivity of polymer solutions. These phenomena can be explained by the net charge density. A higher net charge density can increase the electrical force exerted on the jet and lead to a decrease in the fiber diameter [38,39].

### 2.4. Solid State of ENFs

**Figure 3:**
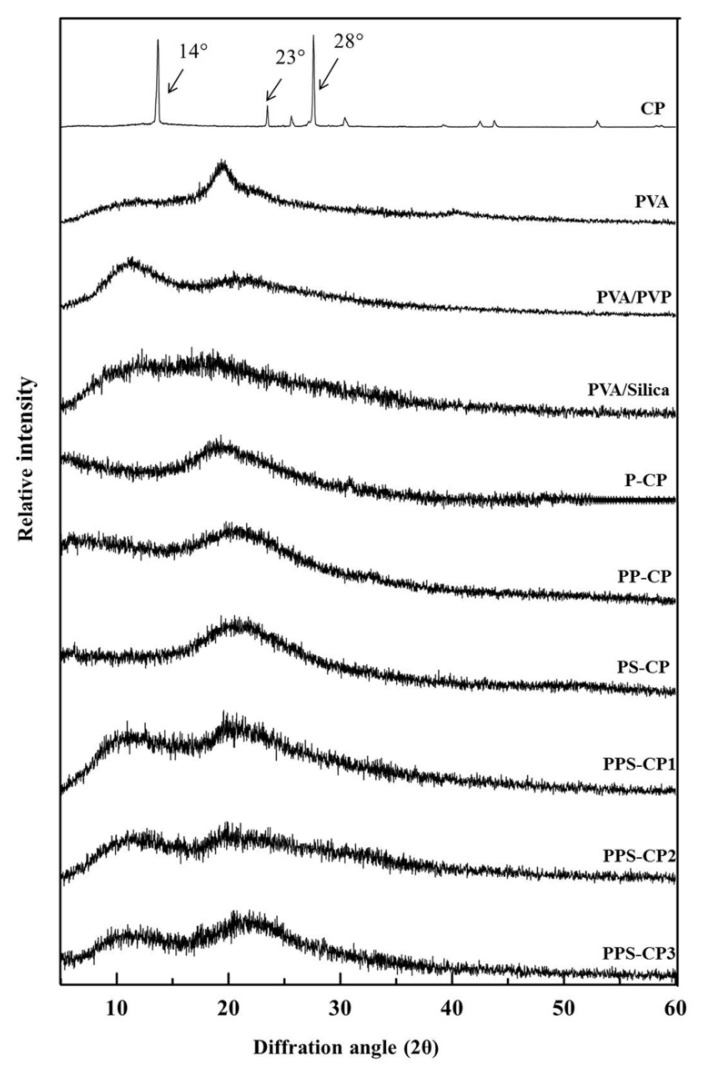
XRD patterns of CP-ENFs and pure excipients in EFASs in comparison with intact crystalline CP.

The X-ray diffraction (XRD) patterns of the different samples are shown in Figure 3. CP is a crystalline material with characteristic X-ray diffraction 2θ angles of 14°, 23°, and 28°. However, the halo pattern is obtained after CP is loaded in the nanofibers. This indicates that CP is dispersed as a non-crystalline form which may be molecular dispersion or amorphous form. This result suggests that during the electrospinning process, the solid fibers were rapidly formed on the collector by removing the solvent instantly. The time of solidification of the nanofibers was too short for the drug to recrystallize. 

### 2.5. Adhesive Property and Entrapment Efficiency of ENFs

Nanofibers with high adhesive property can provide prolonged residence time on the desired site of action and thereby exert a better bleaching effect. Due to the good adhesive properties of PVA and PVP, previous studies have used these polymers in mucoadhesive dosage forms [40,41]. Among the seven samples, ENF from PP-CP possesses the highest adhesive force with a value of 0.81 ± 0.02 N followed by PPS-CP3, PPS-CP1, PPS-CP2, P-CP, and PS-CP, respectively (Table 3). These results suggest that PVP can enhance the adhesive properties of PVA. The result of the present study confirms that these two polymers are suitable as adhesive materials for CP-ENF. It has previously been reported that ENF of polycaprolactone using PVP as an exterior sheath, helped the fibers to rapidly adhere to wet biological surfaces [42]. In the present study, it is observed that CP-ENF containing PVA or a combination of PVA and PVP are non-adhesive under dry conditions but can readily adhere to wet mucosal tissues. This result confirms that the adhesive power of these polymers dramatically increases when they are subjected to a high humidity or moist environment. According to the adhesive properties of PVA and PVP in a wet environment [43], CP-ENFs can strongly adhere to the non-uniform tooth surfaces in the oral cavity surrounded by saliva.

For drug loading capacity, the results show that entrapment efficiency (EE) values of CP-ENFs are in the range of 59–98% depending on the type and concentration of the components in each formulation. EFASs containing various compositions of PVA, PVP, silica, CP, and water (as presented in Table 1) were subjected to the electrospinning process, the water evaporated and ENFs formed, therefore the obtained ENFs contained 90–95% polymer with and without silica and the final concentration of CP in the ENFs ideally was 10% for ENFs obtained from 1% CP concentration EFASs and 5% for ENFs obtained from 0.5% CP concentration EFASs. The actual CP amount in the obtained CP-ENFs from various EFASs was 5.9 ± 0.2%, 7.4 ± 0.1%, 7.7 ± 0.2%, 8.3 ± 0.2%, 8.8 ± 0.1% for P-CP, PP-CP, PS-CP, PPS-CP1, and PPS-CP2, respectively, and 4.9 ± 0.1% for PPS-CP3. Therefore, ENF obtained from P-CP possesses the lowest EE with a value of 60 ± 2%, whereas that from PPS-CP3 demonstrates the highest EE with a value of 98 ± 2%. The lowest EE value of P-CP fibers is due to the instability of CP in P-CP. Decomposition of CP can occur before and during the electrospinning process. Ideally, the amount of drug entrapped in the nanofibers should be equal to the amount of drug added before the electrospinning process. However, CP is unstable, particularly when exposed to oxygen and light. From the stability results mentioned above, it is shown that the highest amount of CP remaining can be found in the solution containing PVP. This result supports that PVP plays an important role on stabilization of CP in EFASs. For the systems containing silica, it is considered that silanol groups of silica can form strong hydrogen bond with CP [29]. Therefore, the highly porous property with large surface area of silica can entrap and protect CP from decomposition, leading to the high drug content in the fibers. The results show that EE values of the silica containing ENFs obtained from PPS-CP1, PPS-CP2, and PPS-CP3 are significantly higher than from P-CP, PP-CP, and PS-CP, respectively (*p* < 0.05). Moreover, it is noted that EE values of ENFs from PPS-CP1, PPS-CP2, and PPS-CP3 are also different. Among them, ENF from PPS-CP3 demonstrates the highest EE value. During the electrospinning process, it was found that the jet streams of PPS-CP1 and PPS-CP2 were broken, resulting in the formation of beaded nanofibers. This phenomenon indicates that composition ratios of PPS-CP1 and PPS-CP2 in EFASs are important. The inappropriate ratio can hardly provide a stable electrospun fibers. Kundrat et al. reported the effects of viscosity on EE values of levofloxacin loaded poly3-hydroxybutyrate nanofibers [44]. Our experiment also shows that EE values of the obtained CP-ENFs are dependent on viscosity of EFASs. It is found that increase in viscosity can affect the formation of a stable jet leading to a difficulty in stable and uniform nanofiber formation since the solution can easily dehydrate at the tip of the needle thereby the components can adhere to the surfaces of the needle and the flow of components through the tip is disrupted. We, therefore, conclude that the composition ratio of EFAS plays an important role on the EE value of the obtained fibers by affecting conductivity and viscosity of EFASs.

### 2.6. In Vitro Drug Release and Drug Release Kinetics

**Figure 4:**
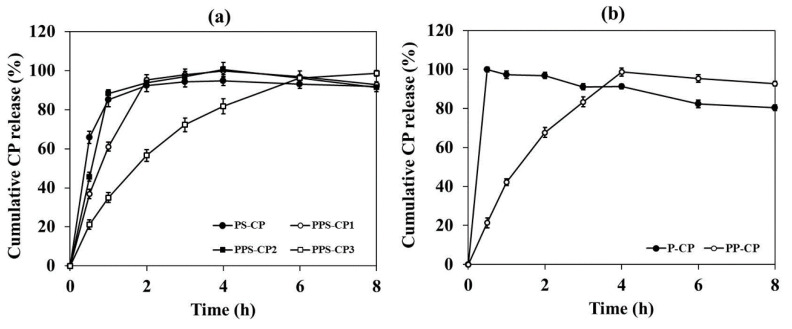
In vitro release of CP from CP-ENFs obtained from various EFASs with (**a**) and without (**b**) silica.

Drug release profiles of CP-ENFs obtained from the developed EFASs with and without silica are presented in Figure 4. A burst release of CP is observed from CP-ENF obtained from P-CP. Almost 100% CP is released into the dissolution medium within 30 min. CP-ENF obtained from PP-CP shows a slower drug release compared to that obtained from P-CP, indicating the effect of PVP on retardation of drug release. However, almost the total amount of CP (98.7 ± 1.8%) is released after 4 h. The influence of silica on drug release can be seen among ENFs from EFASs with different amount of silica. At the release period of 1 h, drug release from CP-ENFs obtained from PVA-PVP-silica core-based ENFs such as PPS-CP1, PPS-CP2, and PPS-CP3 ENF was found to be 61.1 ± 1.2%, 88.3 ± 1.1%, and 35.0 ± 2.0%, respectively, indicating that silica can causes a fast drug release. However, at the same period of 1 h, CP released from CP-ENF obtained from P-CP, a film without silica, is faster than that from PS-CP, a film with silica. This result shows the effect of brittleness and ununiformed drug distribution of the film. During the experiment, it was observed that P-CP film was easily broken into small pieces and dispersed into the dissolution medium, leading to an extreme increase of contact surface area of the film to the medium hence a faster release of the drug can be obtained. Therefore, the effect of silica on increasing drug release from PS-CP film is obscured.

The results from the drug release study also suggest that release of CP from the nanofibers is influenced by the viscosity of EFASs and morphology of the nanofibers. High viscosity can prolong drug release. This can be seen in the release behavior of ENF obtained from EFASs containing PVA and PVP mixture, which possesses high viscosity. However, for the ENF from P-CP, which also possesses high viscosity, a burst release is observed from its fibers. This result might be due to the ununiformed drug dispersion and the destabilization of PVA polymer chains induced by CP, leading to a high amount of drug being located on the fiber surface. The results demonstrate that replacement of silica and PVP to PVA can decrease brittleness of the films and hence the slow drug release can be observed. Among the others, the ENF obtained from PPS-CP3 shows the most sustained drug release within 8 h.

**Figure 5:**
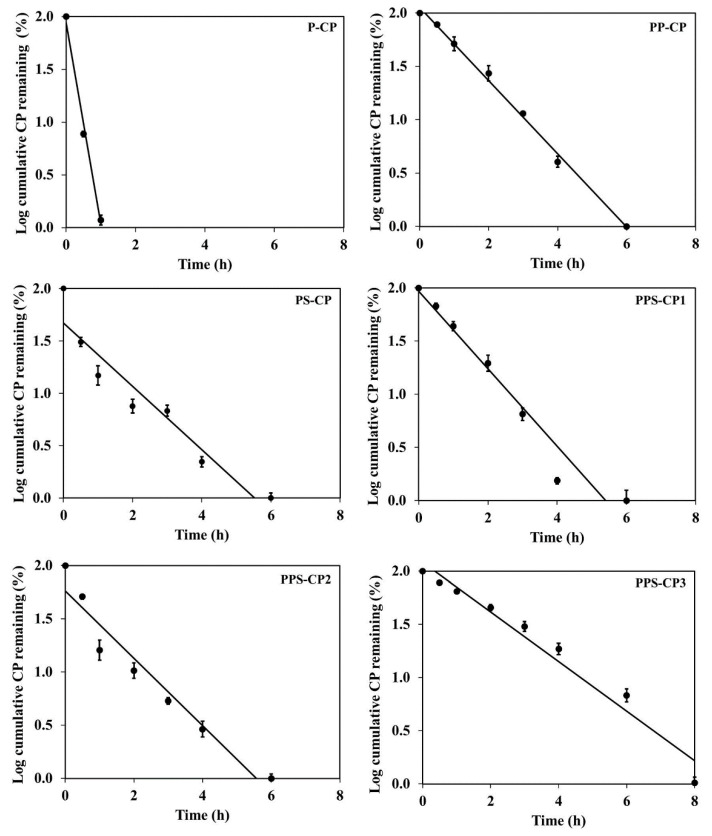
Kinetic plots of CP release according to the first-order kinetics.

Kinetic study of drug release can provide a meaningful parameter to understand the drug release mechanism for the appropriate purpose and the results can be utilized to assess the effect of formulation factors on the drug release profile [45]. Several mathematical models can be used to describe the kinetic drug release from the developed formulations, such as the zero-order and the first-order models [46]. The Korsmeyer–Peppas model has been used to describe the type of drug release mechanism from polymeric systems [47]. In the present study, the release data were analyzed by these kinetic models. The zero-order and the first-order kinetic models are expressed in the following equations,

$$Q_{t}{=Q}_{0}{+k}_{0}t$$

and

$$Q_{t}{=Q}_{0}{+(1-e}^{{-k}_{1}t})$$

respectively, where Q_0_ is initial amount of drug in the solution, k_0_ is the zero-order release rate constant, k_1_ is the first-order release rate constant, and t is time of drug release. A suitable mathematical model is chosen by value of a correlation coefficient (r^2^) from linear regression analysis. The results are reported in Table 4. Comparing the r^2^ values between the zero- and the first-order kinetics, the results described that the release of CP from all CP-ENFs followed the first-order kinetics, which the linear relationship can be obtained as shown in Figure 5, indicating that the release rate of CP is dependent on drug concentration. 

**Figure 6:**
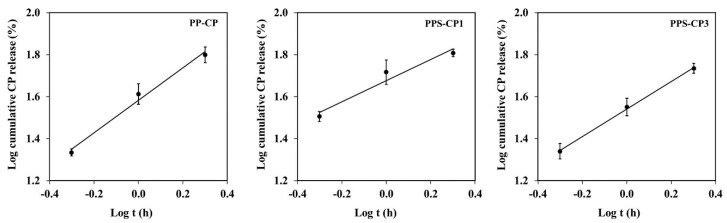
Kinetic plots of CP release according to the Korsmeyer–Peppas model.

Drug release mechanism was analyzed using Korsmeyer–Peppas model, as the following equation

(M_t_/M_∞_) = k_kp_t^n^

where M_t_/M_∞_ is a fraction of drug released at time t, k_kp_ is the release rate constant of Korsmeyer–Peppas, and n is the exponent of release in function of time t which indicates the mechanism of drug release. When n is ≤0.45, the drug release mechanism follows a Fickian diffusion mechanism. When 0.45 < n < 0.89, it follows non-Fickian diffusion or anomalous transport. When n is 0.89, it follows case II transport, and when n is >0.89, it follows super case II transport mechanism [48,49]. In general, Korsmeyer–Peppas model is a mathematical model suitable for sustained release kinetics and the most valid prediction using this model should be performed on M_t_/M_∞_ ≤ 0.6 of drug release [48,50]. Therefore, this model is not suitable for ENFs of P-CP, PS-CP, and PPS-CP2 due to their fast release behavior but may be suitable for ENFs from PP-CP, PPS-CP1, and PPS-CP3 due to their sustained release property. According to the Korsmeyer–Peppas model, log cumulative drug release against log time of these three formulations was plotted as shown in Figure 6. The kinetic parameters evaluated from these graphical plots are shown in Table 4. The correlation coefficient by linear regression analysis which closed to 1 was considered as the fitted model. It is found that the r^2^ values of PP-CP, PPS-CP1, and PPS-CP3 from the Korsmeyer–Peppas model are greater than 0.95. The rate constant of CP-ENF from PPS-CP1 was the highest compared to that from PP-CP and PPS-CP3, possibly due to low amount of PVA. The drug release from CP-ENF from PP-CP, PPS-CP1, and PPS-CP3 is through non-Fickian diffusion as their n values are between 0.45 and 0.89. This indicates anomalous transport which is a combination of Fickian diffusion and case II transport. During these processes, rearrangement of polymeric chains caused by swelling and erosion can be occurred and the diffusion process simultaneously cause the time-dependent anomalous effects [51]. Therefore, the release of CP from ENFs is controlled by the swelling and erosion of ENFs and the diffusion of CP. The results of this experiment also indicate that the different amount of the compositions of ENFs can influence the kinetics of drug release from the films. 

### 2.7. Ex Vivo Tooth Bleaching Efficiency

**Figure 7:**
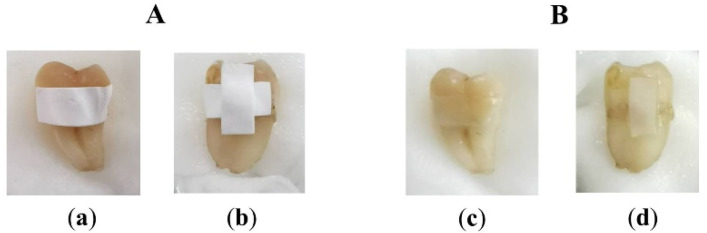
The photographs of tooth sample before (**A**) and after (**B**) 8 h of bleaching test; ENFs in horizontal direction (**a**,**c**) and ENFs in horizontal and vertical directions (**b**,**d**).

The bleaching protocol used in the present study was modified from the dental bleaching treatment recommended by American Dental Association [52]. A period of 8 h bleaching per day is the suggested duration for bleaching formulations having a drug concentration not exceeding 10% (classified as a low drug concentration) [53]. The concentration of CP in all ENF was not higher than 10%, therefore, they are regarding as low concentration formulations [54]. Thus, the protocol with 8 h of bleaching per day was used in the current study with a period of 14 days. ENFs were placed on the tooth surface and the teeth were kept in the controlled environment. After 8 h of bleaching, it was found that the remaining films still were attached on the tooth surface as presented in Figure 7. The films were more transparent and of smaller sizes than before the test.

**Figure 8:**
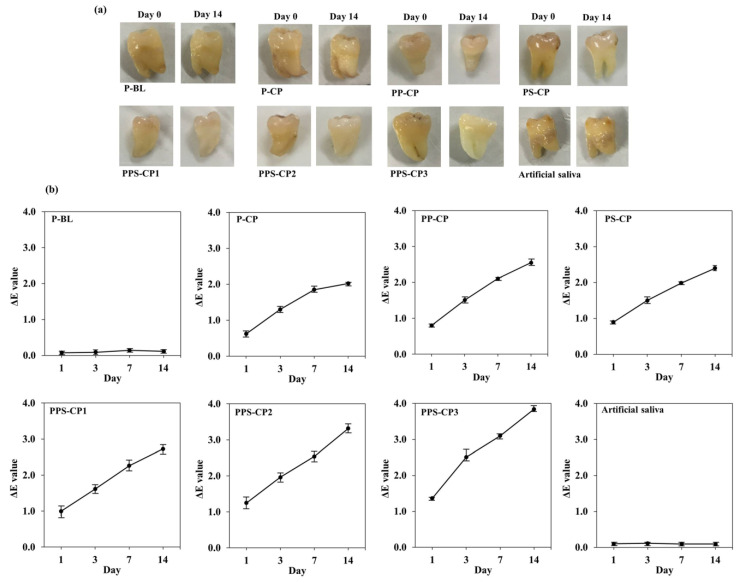
Ex vivo tooth bleaching efficiency of ENFs in comparison with negative control groups; photographs of tooth sample before and after completing the bleaching protocol (**a**) and the mean ΔE values progression for 14 days (**b**).

The results demonstrate that after complete bleaching period of 14 days, the tooth color of the treatment groups, i.e., P-CP, PP-CP, PS-CP, PPS-CP1, PPS-CP2, and PPS-CP3, was whiter than the initial day as seen in Figure 8a. The visual color change (ΔE) value progressions of each formulation are presented in Figure 8b. All CP-ENFs cause a significantly potential bleaching effect as compared to the negative control groups for day 0, day 1, day 3, day 7, and day 14 (*p* < 0.05). It is noted that on day 14, the mean ∆E value of ENF obtained from PPS-CP3 is the highest (*p* < 0.05) among the tested ENFs with a value of 3.84 ± 0.08. Since ENF obtained from PPS-CP3 possesses the highest EE value and high adhesion, this formulation could efficiently serve as a reservoir for drug and increase drug residence time on the teeth. As shown in the in vitro release tests, this formulation provided sustained release profiles of CP, resulting in the enhanced bleaching efficiency of the drug at the site of action.

Current literature indicates that ∆E values from using CP vary due to differences in staining procedure, type of product, duration of application, and the applied amount. It has been reported that ∆E values of at least 2.6 are visually perceptible [55], ∆E values of ENF obtained from PPS-CP3 were 2.51 ± 0.21 at day 3, and of PPS-CP1 and PPS-CP2 were above 2.6 at day 14. Therefore, these formulations were effective for tooth bleaching. However, it was observed that the entire tooth was bleached. A possible explanation for this result is that after CP-ENFs were hydrated by artificial saliva, the nanofibers were swelling, and erosion of the polymer occurred. ENFs transformed into a hydrogel film formulation. CP can diffuse from ENFs and dissolve in saliva that surrounded the teeth, leading to an increase of the contact area. After 8 h, some ENFs showed small parts remaining on the tooth surface whereas most of ENFs were dissolved. Moreover, the placement of ENF obtained from PPS-CP3 was in two directions; therefore, it covers more area of the tooth compared to the others. In addition, it was difficult to control and localize CP delivery especially when teeth not cut into flat surfaces were used. However, this provides an issue in the use of ENFs that needs to be further be explored especially for in vivo study. Further research of CP-ENFs in order to prevent the drug from contacting an unwanted area such as gingival is needed.

Another aspect of CP-ENFs that should be addressed in future studies are the treatment time, as in this study, the protocol was selected based on CP concentration in the formulations without considerations on the dosage form. The developed CP-ENFs have shown satisfactory results of bleaching efficiency in 8 h per day for 14 days. However, commercial products such as strips typically require an application of only 30 min, twice a day, for 14 days [56]. As ENFs are introduced for tooth bleaching here for the first time, the suitable treatment regimen still needs to be investigated in order to compare efficiency with commercial products.

In addition, the obtained ENFs could possibly retard CP degradation compared to hydrophilic formulations, especially for long-term storage. CP-ENFs avoid the use of organic solvents, stabilizers, and other additive substances, such as glycerin which is commonly used in CP gels and may lead to tooth dehydration and sore throat [57,58].

## 3. Materials and Methods

### 3.1. Materials

Hydrophilic fumed silica (Aerosil^®^ 380) was obtained from Evonik (Essen, Germany). CP, PVA having a molecular weight range of 85,000-124,000 and 87–89% degree of hydrolysis, PVP K-90, and triphenylphosphine were from Sigma Aldrich (St. Louis, MO, USA).

### 3.2. Preparation of EFASs

EFASs with different concentrations of CP and major components for core-based nanofibers as shown in Table 1 were prepared as follows. Solutions of PVA or PVA and PVP in distilled water were prepared under constant stirring of 800 rpm at 70 °C for 12 h and then cooled down to room temperature. These solutions were added into a 1% N,N-dimethylformamide solution containing CP or CP and silica. The obtained mixtures were gently stirred at 100 rpm at room temperature to obtained homogenous spinning solutions without air bubbles. For EFASs without CP such as P-BL, the polymer was dissolved in distilled water and then adjusted to weight with the water. 

### 3.3. Stability of CP in EFASs

The freshly prepared CP-containing EFASs were continuously stirred at 100 rpm at 25 °C throughout the experiment. Samples of 1.0 mL were withdrawn periodically at 30 min, 1, 2, 4, 6, and 8 h after preparation. Quantitative analysis of CP from each withdrawn sample was immediately performed using a high-performance liquid chromatography (HPLC).

### 3.4. HPLC Analysis

Quantitative analysis of CP can be done using the oxidation of triphenylphosphine by CP into triphenylphosphine oxide which can be detected in HPLC chromatogram at a different retention time from that of triphenylphosphine. Therefore, decrease of triphenylphosphine or increase of triphenylphosphine oxide can be calculated and converted to the amount of CP. In the current study, the determination of CP was done by quantification of triphenylphosphine oxide. The analysis was carried out using HPLC (DionexTM ChromeleonTM HPLC System, Thermo Fisher Scientific Inc., Waltham, MA, USA) and a reversed phase column (5 µm, 150 × 4.6 mm I.D, YMCTM Pack Pro C18, YMC Co., Ltd., Kyoto, Japan) as the stationary phase. HPLC condition used was according to a method previously described [59] with some modification. Briefly, an exact volume of 1.0 mL of the sample was added to an equal volume of 0.1 M triphenylphosphine and constantly stirred for 2 h at room temperature. The resulted mixture was filtered through a 0.22 μm filter membrane prior to injecting in HPLC with an injected volume of 10 μL. The HPLC mobile phase consisting of acetonitrile (A) and water (B) was used at a flow rate of 1.0 mL/min with gradient conditions as follows: 40% A and 60% B for 6.5 min, 100% A for 10 min, and 40% A and 60% B for 3.5 min. Detection was performed by means of a UV detector at a wavelength of 225 nm. The temperature of HPLC was maintained at 25 °C. Calibration curve was prepared using CP solution at a concentration range of 30–150 μg/mL and the obtained linear standard curve with r^2^ = 0.9995 was used. 

### 3.5. Viscosity and Electrical Conductivity of EFASs

Viscosity of EFASs was measured using a plate-plate Brookfield rheometer (Rheometer R/S-CPS, Middleboro, MA, USA). The gap between the two plates was 1 mm, a shear rate ranging from 1 to 360 s^−1^ was employed. Electrical conductivity of the solutions was measured using a pH-conductivity meter (D-24 Horiba, Kyoto, Japan). The samples were maintained at 25 °C throughout the tests. The measurements were carried out by directly inserting an electrode (9625-10D, 3-in-1 Electrodes, Horiba, Kyoto, Japan) into the samples. The temperature was maintained at 25 °C throughout the tests. 

### 3.6. Fabrication of the ENF

The developed EFASs were loaded into a 2.5 mL syringe connected with a stainless-steel needle (Hamilton 2.5 mL, Model 1005 TLL SYR, Hamilton Metal Hub Needles, Hamilton Company, Bonaduz, Switzerland). The syringe containing EFAS was placed in a syringe pump (Harvard Apparatus Pump 11 Elite Syringe Pumps, Holliston, MA, USA) and horizontally pumped at a flow rate of 10 µL/min. The electrospinning process was performed with an applied voltage of 15 kV provided by a high voltage power supply (FC Series-Glassman High Voltage Regulated DC Power Supplies, High Bridge, Hunterdon, NJ, USA). The ENF was deposited on a stationary metal collector (VWR International, Radnor, PA, USA) covered with aluminum foil. The distance between the syringe tip and the collector plate was 10 cm. The electrospinning process was conducted at room temperature in an enclosed fume hood. After this fabrication process, the CP-containing EFASs could yield CP-ENFs. The solution without CP e.g., P-BL could yield a blank-ENF. All ENFs obtained were stored in a desiccator for further study.

### 3.7. Morphology Study

The micro-structure of the nanofibers was investigated by using an SEM (JEOL JSM-6610LV, Tokyo, Japan) as follows. The obtained ENF were cut into a rectangle shape of approximately 5 mm × 5 mm and placed on a carbon tape. Then, the sample surface was coated with gold for 15 s using a 40-mA sputter coater (JEOL JFC-1100E, Tokyo, Japan). The SEM images were taken at an accelerating voltage of 15 kV with 3000× and 10,000× magnifications. The average diameters of the nanofibers in the obtained ENF were measured from the SEM images of each sample by using Image J software (US National Institutes of Health, Bethesda, MD, USA).

### 3.8. Investigation of Internal Solid State

An XRD was used to investigate the internal solid state of the obtained CP-ENFs in comparison with CP intact and the other excipients used as major components of the core-based ENF. A Miniflex II desktop XRD (Rigaku, Japan) was used and the XRD diffractograms were recorded in continuous mode over a Bragg angle (2θ) range of 5–60° at the scanning rate of 12°/min.

### 3.9. Investigation of Adhesive Property

The adhesive property of the obtained CP-ENFs and blank ENF was investigated using a method previously described [60], with some modification. Briefly, a texture analyzer (TA.XT PlusTexture Analyzer, Surrey, UK) was calibrated with a 5-kg load cell and equipped with a stand before determining the adhesive forces of the samples. The ENF samples were cut into a circular shape with a diameter of 1 cm and wetted with 100 μL of water before fixing on a probe (P 0.5 Perspex, 0.5-inch diameter). A piece of porcine intestinal mucosa having an inner surface of 2 cm × 5 cm was attached to a glass slide and then placed on the stand. An exact amount of 1 mL artificial saliva [61] was dropped on the surface of the mucosa. The probe was lowered to contact the mucosal surface with the contact force of 0.2 N and contact time of 60 s. After that, the probe was pulled up with the rate of 1 mm/s. Once the probe was completely separated from the mucosa, the data were recorded and calculated by Exponent software (Stable Micro Systems, Surrey, UK) to obtain the adhesive force.

### 3.10. Determination of CP in CP-ENFs

The amount of CP entrapped in the obtained CP-ENFs was investigated by dissolving 0.05 g of each CP-ENF in 50 mL deionized water. The solution was subjected to centrifugation (Beckman Avanti 30 High Speed Compact Centrifuge, Beckman Coulter Life Sciences, Indianapolis, IN, USA) at a speed of 10,000 rpm for 15 min. The supernatant was immediately collected and determined for CP by HPLC analysis as described above. The amount of CP entrapped in the CP-ENF, expressed as EE value, was obtained from the following equation

EE (%) = (D_L_/D_A_) × 100

where D_L_ is the amount of CP in the ENF and D_A_ is the amount of CP initially added.

### 3.11. Drug Release Kinetic

The developed CP-ENFs were cut into a rectangular shapes of an approximate area of 1 cm × 5 cm to obtain an average weight of 0.1 g for each film and then placed in a 30 mL artificial saliva solution at a constant stirring rate of 20 rpm and at a controlled temperature of 37 ± 1 °C. Samples of 1.0 mL were withdrawn from the dissolution medium at time intervals of 30 min, 1, 2, 4, and 8 h. Fresh medium with the same volume was added into the dissolution medium after each withdrawal. The amount of CP was determined by HPLC analysis as described above. The percentage cumulative release of CP at time t (Q_T_) is calculated by the following equation.

Q_t_ = (Q_t_/Q_0_) × 100%

where M_T_ is the cumulative amount of CP released in the release medium at time T and M_0_ is the initial amount of drug in the CP-ENF. The obtained results were analyzed for drug release kinetics using a Microsoft Excel 2010.

### 3.12. Ex Vivo Tooth Bleaching Assessment

**Figure 9:**
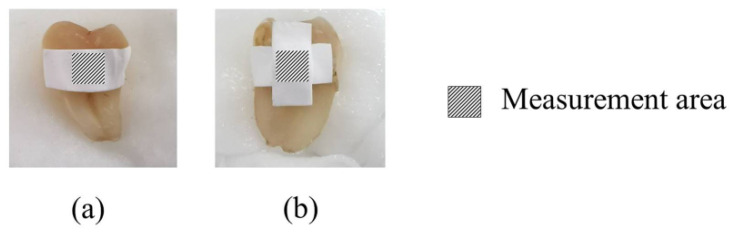
Horizontal placement of the ENFs obtained from P-CP, PP-CP, PS-CP, PPS-CP1, PPS-CP2 (**a**) and horizontal and vertical placements of PPS-CP3 on the tooth surface (**b**).

Human teeth were collected by dentists (Faculty of Dentistry, Chiang Mai University). This study was approved by the Human Experimentation Committee, Faculty of Dentistry, Chiang Mai University (No. 58/2016). The teeth were cleaned and stored in a saturated thymol solution at 4 °C until testing. Forty normal teeth without any surface stains and imperfections were selected. The baseline color at the middle surface of averaged areas of 3 mm^2^ circular centers of the collected teeth was measured. The teeth were randomly allocated into eight groups, five teeth per group, according to the six CP-ENFs obtained from six different CP-containing EFASs and two negative control groups (a blank ENF and artificial saliva). Each day, the fresh ENF samples were cut into rectangle shapes having an area of 5 mm × 10 mm to obtain an average weight of 0.01 g for each film. The obtained films were placed on the enamel surface of the teeth. The CP-ENFs obtained from P-CP, PP-CP, PS-CP, PPS-CP1, and PPS-CP which CP concentration was 1% were placed horizontally whereas those from PPS-CP3 which CP concentration was 0.5%, double films were placed, one in horizontal and the other in vertical directions, as shown in Figure 9, in order to receive the same amount of CP as in CP-ENFs obtained from 1% CP concentration EFASs. After that, the films were wet with 0.05 mL artificial saliva for each tooth and kept in a close container in a controlled temperature of 37 ± 1 °C and relative humidity of 100% for 8 h daily. After 8 h of each day, the films were removed. The teeth were cleaned with distilled water before color measurement. After that, the teeth were stored in the closed containers with sufficient artificial saliva during waiting for the new ENFs on the next day. These procedures were repeated for 14 days.

For tooth color measurement, the color of the teeth was measured by light reflection using a colorimeter (FRU WR10 portable precision colorimeter, Shenzhen Wave Optoelectronics Technology Co., Ltd., Shenzhen, China). Before measurement, the colorimeter was validated for color by means of a spectrocolormeter (UltraScan XE, Hunter Lab, Reston, VA, USA). The scale of the Commission International de l’Eclairage (International Commission on Illumination: CIE) was applied as follows. The measurement reflects three color parameters: L*, a*, and b*. The L* indicates the lightness ranging from black (L* = 0) to white (L* = 100). The a* indicates red-green color, where a positive a* indicating red and a negative a* indicating green color. The parameter b* indicates yellow-blue color, where a positive b* indicates yellow and a negative b* indicates blue color. The ΔE value is often used in order to indicate the perceptible tooth color changes after treatment and can be calculated by using the CIE L*a*b* system and the following equation.

∆E = [(∆L*)^2^ + (∆a*)^2^ + (∆b*)^2^]^½^

where ΔL* = L* baseline − L* after bleaching, Δa* = a* baseline − a* after bleaching, and Δb* = b* baseline − b* after bleaching [62]. The data were collected and calculated for bleaching efficiency evaluation.

### 3.13. Statistical Analysis

Descriptive statistics for continuous variables were calculated and reported as mean ± standard deviation. The analysis of experimental data was performed using SPSS statistics software version 22 with a one-way analysis of variance (ANOVA) and Duncan’s multiple range test (*p* < 0.05).

## 4. Conclusions

The present work is the first study on stabilizing CP by entrapping in the solid state of nanofibrous films using an electrospinning technique. The study demonstrates that the ENF in suitable concentrations of PVA, PVP, and silica is a promising substrate to entrap CP for improving its stability and tooth bleaching activity. PVA mainly acts as a core of the nanofibers while PVP and silica are the important substances for enhancing CP stability and drug loading efficiency of the obtained ENFs. CP, PVP, and silica also affect the viscosity and conductivity of EFASs, and this can influence the morphology and size of the obtained ENFs. The adhesive property of the ENFs is mainly influenced by PVA and PVP. The ENF obtained from PPS-CP3, composed of 0.5% CP, 5.5% PVA, 3% PVP, and 1% silica, is a promising system for CP as it provides good morphology with the highest drug entrapment efficiency and desired controlled release profiles. The release of CP from this CP-ENF is the first-order kinetics. The mechanism of drug release is a non-Fickian diffusion or anomalous transport according to the Korsmeyer–Peppas model. This ENF provides excellent ex vivo tooth bleaching efficiency and is suitable for further in vivo investigation to evaluate the potential efficacy of this innovation.

## Figures and Tables

**Table 1:** Compositions of electrospinning feed aqueous solutions (EFASs).

EFASs	Composition (% *w*/*w*)				
	PVA	PVP	Silica	CP	Water
P-BL	10	-	-	-	90
P-CP	9	-	-	1	90
PP-CP	6	3	-	1	90
PS-CP	8	-	1	1	90
PPS-CP1	5	3	1	1	90
PPS-CP2	4	3	2	1	90
PPS-CP3	5.5	3	1	0.5	90

**Table 2:** Viscosity and conductivity of EFASs.

EFASs	Viscosity (mPas) *	Conductivity (µS/cm) *
P-BL	3.47 ± 0.42 ^a^	1.45 ± 0.05 ^e^
P-CP	2.74 ± 0.11 ^b^	2.13 ± 0.02 ^cd^
PP-CP	3.79 ± 0.20 ^a^	2.43 ± 0.05 ^a^
PS-CP	2.02 ± 0.51 ^c^	2.02 ± 0.03 ^d^
PPS-CP1	2.52 ± 0.40 ^b^	2.24 ± 0.04 ^bc^
PPS-CP2	1.75 ± 0.54 ^c^	2.02 ± 0.04 ^d^
PPS-CP3	3.64 ± 0.40 ^a^	2.32 ± 0.06 ^ab^

* different lowercase letters indicate significant differences (*p* < 0.05).

**Table 3:** Adhesion force and entrapment efficiency (EE) values of ENFs obtained from various EFASs.

EFASs for ENFs	Adhesion Force (N) *	EE (%) *
P-BL	0.77 ± 0.02 ^b^	-
P-CP	0.74 ± 0.02 ^c^	59.48 ± 2.25 ^e^
PP-CP	0.81 ± 0.02 ^a^	73.83 ± 1.37 ^d^
PS-CP	0.69 ± 0.01 ^d^	76.82 ± 1.83 ^d^
PPS-CP1	0.71 ± 0.02 ^cd^	82.67 ± 1.57 ^c^
PPS-CP2	0.63 ± 0.01 ^e^	88.25 ± 1.01 ^b^
PPS-CP3	0.73 ± 0.02 ^c^	98.32 ± 1.87 ^a^

* different lowercase letters indicate significant differences (*p* < 0.05).

**Table 4:** Release kinetics parameters from different kinetic models.

EFASs for ENFs	Release Kinetics						
	Zero Order		First Order		Korsmeyer–Peppas		
	r^2^	k_0_	r^2^	k_0_	r^2^	k_kp_	n
P-CP	0.78	100.29	0.99	6.78	-	-	-
PP-CP	0.94	24.29	0.95	0.95	0.97	1.63	0.86
PS-CP	0.57	17.44	0.86	0.70	-	-	-
PPS-CP1	0.90	24.23	0.94	0.84	0.96	1.75	0.58
PPS-CP2	0.64	19.62	0.90	0.85	-	-	-
PPS-CP3	0.86	11.58	0.95	0.56	0.98	1.54	0.72

## References

[1] Féliz-Matos L., Hernández L.M., Abreu N. (2014). Dental bleaching techniques; hydrogen-carbamide peroxides and light sources for activation, an update. mini review article. Open Dent. J..

[2] Pędziwiatr P., Mikołajczyk F., Zawadzki D., Mikołajczyk K., Bedka A. (2018). Decomposition of hydrogen peroxide—kinetics and review of chosen catalysts. Acta Innov..

[3] Hattab F.N., Qudeimat M.A., Al-Rimawi H.S. (1999). Dental discoloration: An overview. J. Esthet. Restor. Dent..

[4] Dahl J.E., Pallesen U. (2003). Tooth bleaching—A critical review of the biological aspects. Crit. Rev. Oral Biol. Med..

[5] Joiner A. (2006). The bleaching of teeth: A review of the literature. J. Dent..

[6] Rezende M., Ferri L., Kossatz S., Loguercio A.D., Reis A. (2016). Combined bleaching technique using low and high hydrogen peroxide in-office bleaching gel. Oper. Dent..

[7] Tredwin C.J., Naik S., Lewis N.J., Scully C. (2006). Hydrogen peroxide tooth-whitening (bleaching) products: Review of adverse effects and safety issues. Br. Dent. J..

[8] Barcellos D.C., Benetti P., Fernandes V.V.B., Valera M.C. (2010). Effect of carbamide peroxide bleaching gel concentration on the bond strength of dental substrates and resin composite. Oper. Dent..

[9] Meireles S.S., Fontes S.T., Coimbra L.A.A., Bona Á.D., Demarco F.F. (2012). Effectiveness of different carbamide peroxide concentrations used for tooth bleaching: An in vitro study. J. Appl. Oral Sci..

[10] Soares D.G., Basso F.G., Hebling J., de Souza Costa C.A. (2014). Concentrations of and application protocols for hydrogen peroxide bleaching gels: Effects on pulp cell viability and whitening efficacy. J. Dent..

[11] Jing X., Zhibing Z. (2009). Physical, structural, and mechanical characterization of calcium–shellac microspheres as a carrier of carbamide peroxide. J. Appl. Polym. Sci..

[12] Adam P., Sasikanth K., Nama S., Suresh S., Brahmaiah B. (2013). Nanofibers—A new trend in nano drug delivery. Pharma Innov. J..

[13] Lee H.J., Lee S.J., Uthaman S., Thomas R.G., Hyun H., Jeong Y.Y., Cho C.S., Park I.K. (2015). Biomedical applications of magnetically functionalized organic/inorganic hybrid nanofibers. Int. J. Mol. Sci..

[14] Shih Y.V., Chen C., Tsai S., Wang Y.J., Lee O.K. (2006). Growth of mesenchymal stem cells on electrospun type I. Stem Cells.

[15] Barnes C.P., Sell S.A., Boland E.D., Simpson D.G., Bowlin G.L. (2007). Nanofiber technology: Designing the next generation of tissue engineering scaffolds. Adv. Drug Deliv. Rev..

[16] Rieger K.A., Birch N.P., Schiffman J.D. (2013). Designing electrospun nanofiber mats to promote wound healing-a review. J. Mater. Chem. B.

[17] Leung V., Ko F. (2011). Biomedical applications of nanofibers. Polym. Adv. Technol..

[18] Bajakova J., Chaloupek J., Lukaš D., Lacarin M. (2011). Drawing—the production of individual nanofibers by experimental method. Int. Conf. Nanomater. Res. Appl..

[19] Feng L., Li S., Li H., Zhai J., Song Y., Jiang L., Zhu D. (2002). Super-hydrophobic surface of aligned polyacrylonitrile nanofibers. Angew. Chemie Int. Ed..

[20] Katsogiannis K.A.G., Vladisavljević G.T., Georgiadou S. (2015). Porous electrospun polycaprolactone (PCL) fibres by phase separation. Eur. Polym. J..

[21] Rolandi M., Rolandi R. (2014). Self-assembled chitin nanofibers and applications. Adv. Colloid Interface Sci..

[22] Ramakrishna S., Fujihara K., Teo W.E., Yong T., Ma Z., Ramaseshan R. (2006). Electrospun nanofibers: Solving global issues. Mater. Today.

[23] Bhardwaj N., Kundu S.C. (2010). Electrospinning: A fascinating fiber fabrication technique. Biotechnol. Adv..

[24] Hu X., Liu S., Zhou G., Huang Y., Xie Z., Jing X. (2014). Electrospinning of polymeric nano fibers for drug delivery applications. J. Control. Release.

[25] Gaaz T.S., Sulong A.B., Akhtar M.N., Kadhum A.A.H., Mohamad A.B., Al-Amiery A.A. (2015). Properties and applications of polyvinyl alcohol, halloysite nanotubes and their nanocomposites. Molecules.

[26] Huang S., Zhou L., Li M.C., Wu Q., Kojima Y., Zhou D. (2016). Preparation and properties of electrospun poly (vinyl pyrrolidone)/cellulose nanocrystal/silver nanoparticle composite fibers. Materials (Basel).

[27] Nasouri K., Shoushtari A.M., Mojtahedi M.R.M. (2015). Effects of polymer/solvent systems on electrospun polyvinylpyrrolidone nanofiber morphology and diameter. Polym. Sci. Ser. A.

[28] Panarin E.F., Kalninsh K.K., Pestov D.V. (2001). Complexation of hydrogen peroxide with polyvinylpyrrolidone: Ab initio calculations. Eur. Polym. J..

[29] Kovačič B., Vrečer F., Planinšek O. (2011). Solid dispersions of carvedilol with porous silica. Chem. Pharm. Bull..

[30] Sun Y., Cheng S., Lu W., Wang Y., Zhang P., Yao Q. (2019). Electrospun fibers and their application in drug controlled release, biological dressings, tissue repair, and enzyme immobilization. RSC Adv..

[31] Zhong L., Lee B., Yang S. (2018). Establishing vadose zone slow-release carbon sources for enhanced bioremediation using silica suspension. Vadose Zone J..

[32] Sokolowska D., Dziob D., Gorska U., Kieltyka B., Moscicki J.K. (2013). Electric conductivity percolation in naturally dehydrating, lightly wetted, hydrophilic fumed silica powder. Phys. Rev. E Stat. Nonlinear Soft Matter Phys..

[33] Hamad D., Dhib R., Mehrvar M. (2016). Effects of hydrogen peroxide feeding strategies on the photochemical degradation of polyvinyl alcohol. Environ. Technol..

[34] Zhang S.J., Yu H.Q. (2004). Radiation-induced degradation of polyvinyl alcohol in aqueous solutions. Water Res..

[35] Bates J.S., Shanks R.A. (1979). Hydrogen peroxide oxidation of poly(vinyl alcohol). J. Polym. Sci. Polym. Chem. Ed..

[36] Chuangchote S., Sagawa T., Yoshikawa S. (2009). Electrospinning of poly(vinyl pyrrolidone): Effects of solvents on electrospinnability for the fabrication of poly(*p*-phenylene) and TiO_2_ nanofiber. J. Appl. Polym. Sci..

[37] Wasim M., Sabir A., Shafiq M., Jamil T., Thomas S., Pasquini D., Leu S., Gopakuma D. (2019). Electrospinning: A fiber fabrication technique for water purification. Nanoscale Materials in Water Purification.

[38] Shahreen L., Chase G.G. (2015). Effects of electrospinning solution properties on formation of beads in TiO_2_ fibers with PdO particles. J. Eng. Fiber. Fabr..

[39] Beachley V., Wen X. (2009). Effect of electrospinning parameters on the nanofiber diameter and length. Mater. Sci. Eng. C.

[40] Jin B.Z., Dong X.Q., Xu X., Zhang F.H. (2018). Development and in vitro evaluation of mucoadhesive patches of methotrexate for targeted delivery in oral cancer. Oncol. Lett..

[41] Upendra K., Siddarth D. (2010). Design and development of felodipine buccal mucoadhesive patches. Int. J. Curr. Pharm. Res..

[42] Yoon Y.E., Im B.G., Kim J.S., Jang J.H. (2017). Multifunctional self-adhesive fibrous layered matrix (FiLM) for tissue glues and therapeutic carriers. Biomacromolecules.

[43] Lee J. (2005). Intrinsic adhesion properties of poly(vinyl pyrrolidone) to pharmaceutical materials: Humidity effect. Macromol. Biosci..

[44] Kundrat V., Cernekova N., Kovalcik A., Enev V., Marova I. (2019). Drug release kinetics of electrospun PHB meshes. Materials (Basel).

[45] Barzegar-Jalali M., Adibkia K., Valizadeh H., Shadbad M.R., Nokhodchi A., Omidi Y., Mohammadi G., Nezhadi S.H., Hasan M. (2008). Kinetic analysis of drug release from nanoparticles. J. Pharm. Pharm. Sci..

[46] Costa P., Lobo J.M.S. (2001). Modeling and comparison of dissolution profiles. Eur. J. Pharm. Sci..

[47] Dash S., Murthy P.N., Nath L., Chowdhury P. (2010). Kinetic modeling on drug release from controlled drug delivery systems. Acta Pol. Pharm. Drug Res..

[48] Korsmeyer R.W., Gurny R., Doelker E., Buri P., Peppas N.A. (1983). Mechanisms of solute release from porous hydrophilic polymers. Int. J. Pharm..

[49] Peppas N.A., Sahlin J.J. (1989). A simple equation for the description of solute release. III. Coupling of diffusion and relaxation. Int. J. Pharm..

[50] Ritger P.L., Peppas N.A. (1987). A simple equation for description of solute release I. Fickian and non-Fickian release from non-swellable devices in the form of slabs, spheres, cylinders or discs. J. Control. Release.

[51] Bruschi M.L., Bruschi M.L. (2015). Mathematical models of drug release. Strategies to Modify the Drug Release from Pharmaceutical Systems.

[52] ADA Council on Scientific Affairs (2009). Tooth Whitening/Bleaching: Treatment Considerations for Dentists and Their Patients.

[53] Alqahtani M.Q. (2014). Tooth-bleaching procedures and their controversial effects: A literature review. Saudi Dent. J..

[54] Bizhang M., Chun Y.H.P., Damerau K., Singh P., Raab W.H.M., Zimmer S. (2009). Comparative clinical study of the effectiveness of three different bleaching methods. Oper. Dent..

[55] Matis B.A., Cochran M.A., Eckert G. (2009). Review of the effectiveness of various tooth whitening systems. Oper. Dent..

[56] Oliveira G.M., Miguez P.A., Oliveira G.B., Swift E.J., Farrell S., Anastasia M.K., Conde E., Walter R. (2013). Safety and efficacy of a high-adhesion whitening strip under extended wear regimen. J. Dent..

[57] Walsh L.J. (2000). Safety issues relating to the use of hydrogen peroxide in dentistry. Aust. Dent. J..

[58] Joshi S. (2016). An overview of vital teeth bleaching. J. Interdiscip. Dent..

[59] Kaewpinta A., Khongkhunthian S., Chaijareenont P., Okonogi S. (2018). Preparation and characterization of rice gels containing tooth bleaching agent. Drug Discov. Ther..

[60] Tonglairoum P., Ngawhirunpat T., Rojanarata T., Panomsuk S., Kaomongkolgit R., Opanasopit P. (2015). Fabrication of mucoadhesive chitosan coated polyvinylpyrrolidone/cyclodextrin/clotrimazole sandwich patches for oral candidiasis. Carbohydr. Polym..

[61] Dionysopoulos D., Strakas D., Koliniotou-Koumpia E., Koumpia E. (2017). Effect of Er,Cr:YSGG laser irradiation on bovine enamel surface during in-office tooth bleaching ex vivo. Odontology.

[62] Borges B.C.D., Borges J.S., de Melo C.D., Pinheiro I.V.A., dos Santos A.J.S., Braz R., Montes M.A.J.R. (2011). Efficacy of a novel at-home bleaching technique with carbamide peroxides modified by CPP-ACP and its effect on the microhardness of bleached enamel. Oper. Dent..

